# Supplementary material for: The impact of COVID-19 control measures on social contacts and transmission in Kenyan informal settlements
Source: BMC Med. 2020 Oct 5;18:316. doi: 10.1186/s12916-020-01779-4 (PMC7533154; doi:10.1186/s12916-020-01779-4)
Supplement: Supplementary file 2 — Additional file 2. Measurement of socioeconomic status, and food and economic security. [file 12916_2020_1779_MOESM2_ESM.docx]

# Additional file 2: Measurement of socioeconomic status, and food and economic security

The following characteristics of respondents’ households were used in a principle components analysis to categorise the sample into five wealth quintiles. This categorisation was conducted on the full sample of the of the knowledge, attitudes and perceptions (KAP) survey (n=1750), of which 213 completed the in-depth contacts survey in addition:

1. Piped water supplying dwelling or compound
2. Flushing toilet, to sewer, tank, pit latrine, or elsewhere
3. Toilet not shared with other households
4. Cooking fuel of kerosene, natural gas/LPG, or biogas
5. Finished floor (parquet/polished floor, vinyl/asphalt strips, ceramic tiles, cement, or carpet)
6. Concrete or tiles used in roof
7. Dwelling of more than one room
8. Electricity supply to dwelling or compound
9. Television in household
10. Mobile phone in household

Table S2:1 shows the distribution of respondents by socioeconomic quintile. The distribution of respondents across SES quintiles was similar in the KAP survey and the subset of respondents who were randomly chosen to complete the contacts survey.

| Quintile | Respondents (contacts survey) | % | Respondents (KAP survey) | % |
| --- | --- | --- | --- | --- |
| 1 (=poorest) | 36 | 17 | 362 | 21 |
| 2 | 47 | 22 | 343 | 20 |
| 3 | 40 | 19 | 351 | 20 |
| 4 | 71 | 33 | 553 | 32 |
| 5(=richest) | 19 | 9 | 141 | 8 |
| **Total** | 213 |  | 1750 |  |

**Table S2:1: Breakdown of socioeconomic quintiles in samples**

## Impact on food and economic security

Participants were asked the following questions to elicit the implications of COVID-19 and control measures on food and economic security:

*I want to ask a few more questions about how the Coronavirus pandemic, and the responses of the government and others to try prevent the spread of Coronavirus,’ may have affected you. Your responses will not have an effect on anything you may receive, so please answer as honestly as possible. In the past two weeks, have you experienced any of the following as compared to before the Coronavirus started?*

- *See my family less*
- *See my friends less*
- *Avoid public transport*
- *Complete loss of job/income*
- *Partial loss of job/income*
- *Increased expenses for the household*
- *More housework (cooking, cleaning, caring for children/sick)*
- *More tensions in the household*
- *Increase of crime in your neighborhood?*
- *Experienced more violence outside the house?*
- *Experienced more violence inside the household?*
- *Not purchasing sanitary pads (women only)*
- *Not accessing health care/services/medicines that you would have otherwise needed*
- *Increase in food prices*
- *Other*
