# Supplementary material for: The impact of COVID-19 control measures on social contacts and transmission in Kenyan informal settlements
Source: BMC Med. 2020 Oct 5;18:316. doi: 10.1186/s12916-020-01779-4 (PMC7533154; doi:10.1186/s12916-020-01779-4)
Supplement: Supplementary file 3 — Additional file 3. Age adjustment. [file 12916_2020_1779_MOESM3_ESM.docx]

# Additional file 3: Age adjustment

The mixing matrices of Kiti et al. (2014) [21] and Prem et al. (2017) [19] and (2020) [20] were adjusted for the informal settlement age-distribution. We obtained Kenyan country-level age distribution data from the UN World Population Prospects for 2020. We obtained sub-county age distributions for Kilifi and informal settlements from 2019 Kenyan census data[33]. Datasets are available in the study [github repository.](https://github.com/mquaife/kenya_mixing)


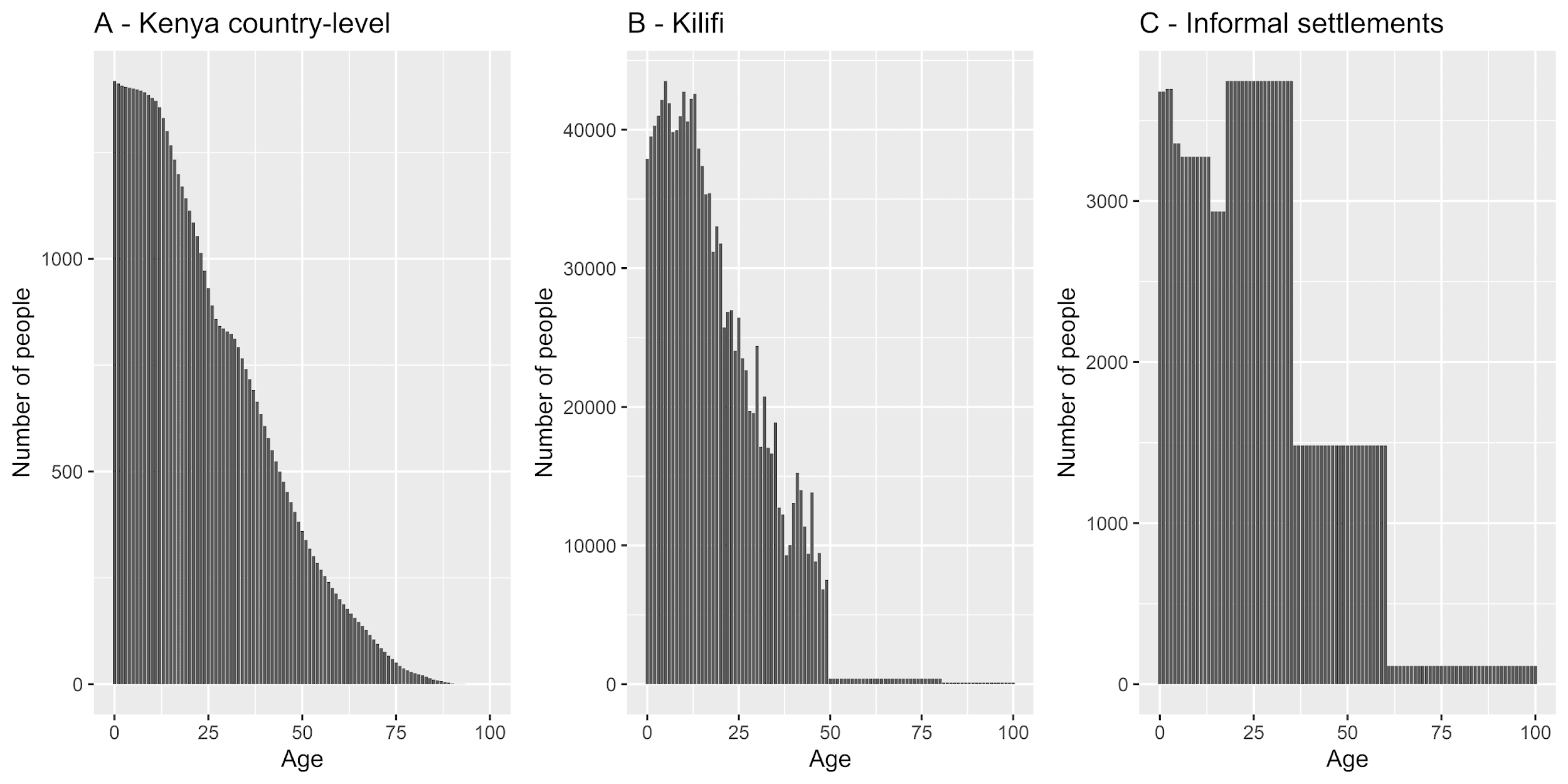


**Figure S3:1 – Age distributions for (A) Kenya country-level, (B) Kilifi, and (C) Nairobi informal settlements**
