# Supplementary material for: The impact of COVID-19 control measures on social contacts and transmission in Kenyan informal settlements
Source: BMC Med. 2020 Oct 5;18:316. doi: 10.1186/s12916-020-01779-4 (PMC7533154; doi:10.1186/s12916-020-01779-4)

# Additional file 4: Age-susceptibility adjustment

Figure S4:1 shows estimates of R_0_ accounting for evidence that children (under 18s) are 50% less susceptible to SARS-CoV2 acquisition[26].


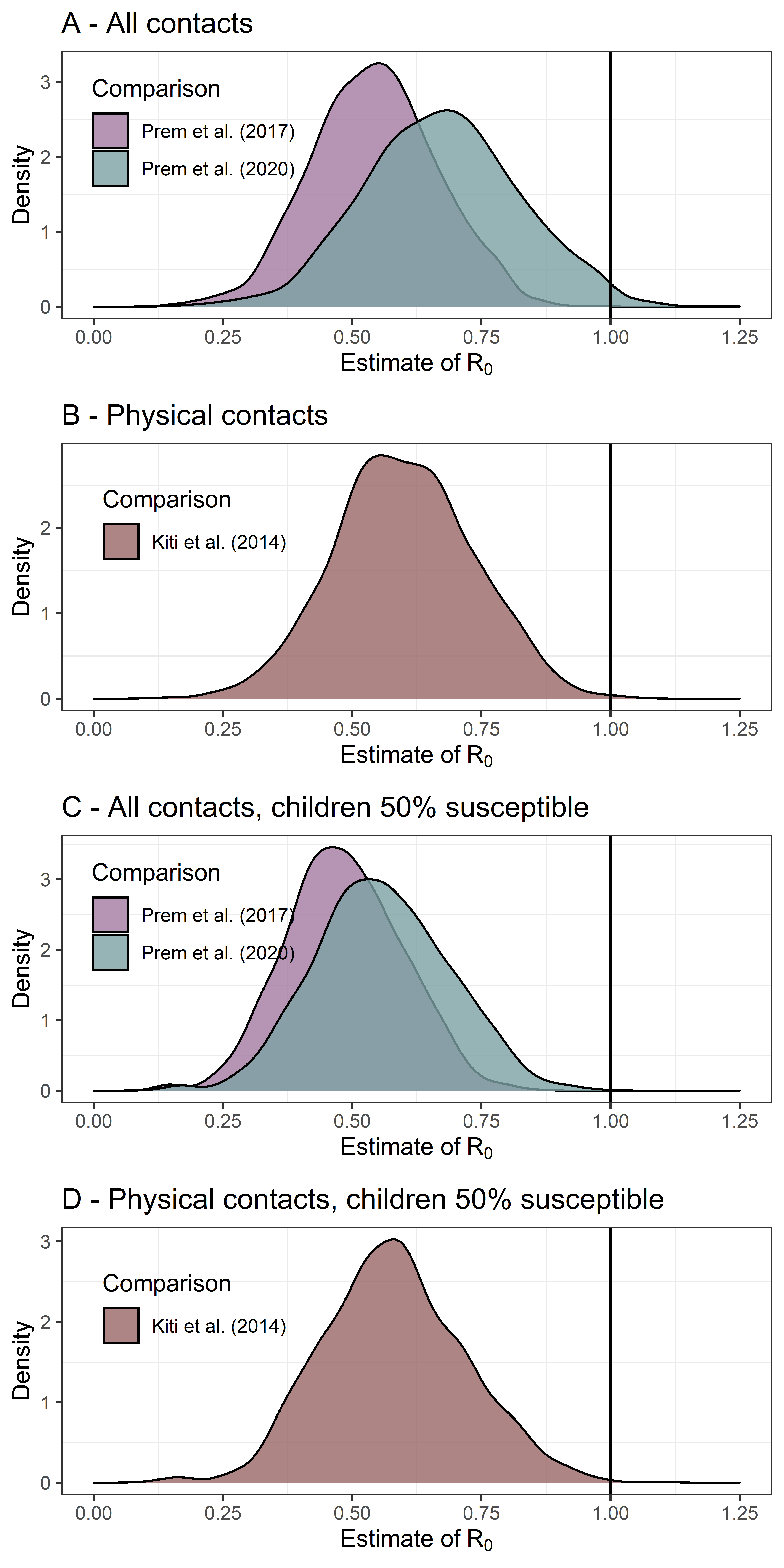

Supplement: Supplementary file 4 — Additional file 4. Age-susceptibility adjustment. [file 12916_2020_1779_MOESM4_ESM.docx]
